# A review of the relationship between mental health, wellbeing and drought resilience in rural, regional and remote communities

**DOI:** 10.3389/fpsyt.2025.1528573

**Published:** 2025-05-26

**Authors:** Emma K. Austin, Anthony S. Kiem

**Affiliations:** Centre for Water, Climate and Land (CWCL), College of Engineering, Science and Environment (CESE), University of Newcastle, Callaghan, NSW, Australia

**Keywords:** drought, resilience, mental health, wellbeing, rural, interventions

## Abstract

Rural, regional and remote communities are vulnerable to the impacts of drought due to their reliance on agriculture, water intensive industries and nature-based tourism. These communities experience significant environmental, social, economic and health impacts because of the reoccurring nature of droughts. This paper reviews the literature on the relationship between mental health, wellbeing and drought resilience and finds that increasingly, mental health and wellbeing are being addressed through a range of drought resilience interventions as it is recognised that good (poor) mental health and wellbeing is linked to high (low) drought resilience. This is because improving mental health and wellbeing increases adaptive capacity and high adaptive capacity is necessary for high drought resilience. Despite this recent progress it is also recognised that there is a lack of tools and resources that (a) recognise when people need help and (b) efficiently connect people to help that is available at the time the help is required. These findings will inform the development of an online rural wellbeing toolkit aimed at enhancing mental health and wellbeing in order to improve drought-resilience in rural, regional and remote Australian communities.

## 1 Introduction

The environmental, social, economic and health impacts of drought are catastrophic (1–4). A pervasive and recurring element of the global climate, drought causes a range of direct and indirect impacts to communities. While the public health impacts of drought are well recognised (5), effects to mental health and wellbeing (MHWB) in particular are increasingly receiving attention (6). Changes in the global climate indicate future droughts may be more frequent, severe and longer in duration (7).

Drought is an inherent part of the Australian climate; therefore, understanding how to increase drought-resilience is imperative. A 2022 report of farmer and community perceptions of drought, found that droughts wear down community resilience, leaving communities exposed and vulnerable to subsequent climate events such as floods and bushfires (8). However, it is not always the case that drought erodes resilience, whereby communities with effective strategies in place can indeed prosper despite drought. For example, resilience is enhanced in an agricultural drought management context when drought risk is controlled prior to a drought event (proactivity) and with local strategies and collaboration (suitability) (9). In another example, resilience was boosted by a women in drought affected Queensland, Australia, through the use of entrepreneurial bricolage, where new problems and opportunities are tackled with the creative use of available resources (10). Overall, MHWB is associated with stronger adaptive capacity (11), suggesting that improved MHWB can lead to increased drought resilience.

This paper has two objectives: (i) summarise the literature focused on drought resilience and MHWB in rural, regional and remote (RRR) Australia; and (ii) document examples of drought resilience interventions aimed at improving MHWB in RRR locations in New South Wales (NSW) and Queensland (QLD), Australia. Findings will inform the development of a co-designed online rural wellbeing toolkit, one of the 14 activities identified as priorities to be addressed by the Southern Queensland and Northern New South Wales (SQNNSW) Drought Resilience Adoption and Innovation Hub (herein “the Hub”). The Hub (www.unisq.edu.au/research/sqnnsw-hub) is one of eight national Drought Resilience Adoption and Innovation Hubs funded by the multi-billion-dollar Future Drought Fund (www.agriculture.gov.au/agriculture-land/farm-food-drought/drought/future-drought-fund). The Hub aims to promote and maintain research, development, extension, adoption, commercialisation and knowledge to help communities thrive in the face of drought.

## 2 Methods

This scoping review includes peer-reviewed and grey literature sourced using the following methods informed by Arksey and O’malley (12). A systematic approach was used to conduct searches in academic databases from the fields of hydrology and climatology, health, and environmental studies. Databases searched include PubMed, Scopus and Google Scholar. Results from these searches were limited to articles in English. Articles from China, Africa and other non-Organisation for Economic Co-operation and Development (OECD) nations were excluded as these countries have a different context of drought resilience, and do not represent the Australian experience. Articles were also sourced from reference lists, conferences, and journal Table of Contents. Relevant government, private organisation and non-government organisations (NGO) websites were also searched. All search results were stored and catalogued in Endnote. The keywords searched from January 2000 to August 2024 were: “wellbeing OR well being OR well-being OR mental health” AND “drought” AND “resilience” AND intervention AND Australia. Boolean operators and truncation symbols were used.

Community consultation, conducted by the authors as part of previous projects (11, 13) steered the direction of the literature review and informed the selection of drought resilience intervention examples included in this paper. Community consultation across several projects included, but was not limited to, stakeholder workshops and focus groups, one-on-one interviews, online surveys, and attendance at networking events, forums and conferences. A range of stakeholders participated in consultations including community service workers, Local, State and Federal Government representatives from several departments, farmers, community leaders, and charitable organisations. Interventions were also sourced from Google searches and grey literature from NGOs, Not-For-Profits (NFPs) and industry advocacy organisations.

## 3 Definitions

This section provides context to the key terms used throughout, and highlights that many (e.g. drought, MHWB and resilience) are not conclusively defined in the literature or policy. For the purpose of this paper, RRR communities are defined as areas outside major cities as per the Australian Standard Geographical Classification – Remoteness Areas (14).

Defining drought is the focus of much research and literature. Despite, or indeed perhaps because of, the widespread occurrence of drought globally, there is no universal agreement on the conceptualisation or definition of drought (3). Labelled as a ‘creeping disaster’, determining both onset and cessation of drought is challenging (15). In simplest terms, drought is typically defined by an extreme and prolonged deficit in precipitation (16, 17) over a specific timeframe (18) and in a specific region (19). However, as our understanding of drought has increased (3, 20–23) along with acknowledgement that drought is more than a lack of rainfall – many different drought definitions have emerged to distinguish between meteorological drought, soil moisture (or agricultural) drought, ecological drought, hydrological or water resources drought, and socioeconomic drought.

As with drought, there is no universally accepted definition for MHWB. The World Health Organisation (WHO) definition is commonly used, describing the link between MHWB as:

“Mental health is a state of wellbeing in which an individual realizes his or her own abilities, can cope with the normal stresses of life, can work productively and is able to make a contribution to his or her community.” (24)

In this review, we define MHWB according to the framework used by the Australian Institute of Health and Welfare (AIHW) which has seven domains: health, education and skills, home, safety, economic, empowerment, and social and community (25). Therefore, MHWB encompasses positive mental health and psychological distress, as well as variables such as physical health, quality of relationships, satisfaction with community and satisfaction with life. Importantly, MHWB is influenced by a range of factors including financial position, isolation, marital status, age, employment and education (26).

The term resilience is used widely in academic literature and Australian public policy. However, Reid and Botterill (27) warn the use of resilience is ambiguous and exposed to varied interpretations when used in public policy debate and, despite its long-term use in some disciplines, definitions are no clearer in academia. For the purpose of this review, resilience is defined as the capacity for individuals and communities to plan for, and cope with, stresses and shocks, adapt to those changes, and have a transformational response (28). Resilience is viewed as encompassing biophysical, sociocultural and economic dimensions, occurring on multiple scales, with key cultural and collaborative components (29). In addition, resilience is recognised as a collection of resources including equity, social capital, communication and information that facilitate the ability to cope with adversity, including drought (30). The definition of drought resilience interventions is broad, to encompass the varied set of diverse and interconnected strategies occurring in RRR communities (31).

## 4 Drought resilience and MHWB in Australian RRR communities

Studies that investigate drought and MHWB are predominantly Australian; 52 of 82 papers included in a seminal systematic review were Australian (32). There is a considerable body of work on impacts related to climatic extremes, including empirical studies on drought, that are both quantitative and qualitative and use different measures to investigate drought, and individual and community wellbeing. Lester et al. (33) describes the range of direct and indirect impacts of drought on MHWB, including employment and education, family relationships, population change, lack of trust in government, and uncertainty about the future.

Buikstra et al. (34) identified eleven components of resilience in Australian RRR communities: social networks and support, positive outlook, learning, early experience, environment and lifestyle, infrastructure and support services, sense of purpose, diverse and innovative economy, embracing differences, beliefs, and leadership. Resilience concepts are increasingly implemented globally in disaster risk reduction, however challenges remain. Wither et al. (35) identified that addressing loss of nuance, clarifying the underlying normative dimensions of resilience, and enhancing social and human capitals, may strengthen the contribution of resilience thinking and lead to more successful outcomes. To deliver successful drought resilience policy, understanding of drought risk and resilience must be integral and include associated uncertainties and caveats (36).

Vulnerability and drought resilience differ across locations and populations, with sociodemographics, health and financial position contributing to an individual’s adaptive capacity and ability to cope with drought (37). Differences in drought vulnerability and mental health due to gender and age are established (38, 39). Hanigan et al. (37) further explored these factors, together with farming status, and found distinct and complex relationships of vulnerability and mental health between subgroups. For farmers who experienced a recent stressful event during protracted drought, psychological and behavioural coping strategies were found to impact distress (40).

RRR communities are typically the worst affected by drought as many are heavily reliant on primary production and water intensive industries to maintain economic viability which increases their vulnerability, and significantly restricts options for adapting to drought (13). Drought is a threat multiplier; it exacerbates and amplifies existing social, political, economic, environmental, health, and ecological stressors in RRR communities. Stressors in RRR communities, such as shifting demographics, the availability of personal and community support services, and the strength of social networks, can exacerbate or buffer the impacts of drought, resulting in changes to adaptive capacity. Understanding the factors that impact adaptive capacity, such as MHWB, is essential when trying to implement successful adaptation strategies (11).

Research that investigates the impacts of drought often focuses specifically on impacts to farmers (41). While farmers do bear much of the burden of drought in many ways, it is apparent that the whole RRR community suffers under drought via shifting demographics, loss of economic activity, and disruptions to community networks (42). The two groups do not exist in isolation; they rely heavily on each other for survival, existing in symbiotic balance (43). A holistic view of RRR communities is essential when considering drought and to leave RRR residents who are not farmers out of the conversation could be detrimental to the usefulness of findings.

Farmers experience a unique set of stressors that increase their risk of MHWB problems, including isolation, long work hours, climate and drought, uncertainty about the future and market shifts (44, 45). In addition, declining communities, increasing rates of change (46), rising inflation and global competition (47) are compounding stressors for farmers. Also, experiences of drought may differ according to type of crop or livestock (48). Financial hardship is well documented as a risk factor for farmers with a consistent and significant relationship between lower income and poor MHWB (49). Experiences of drought are also influenced by gender and age (47, 50, 51). In addition to these occupational factors, farmers are exposed to unique barriers to help-seeking, such as being stoic and in control, as well as limited understanding of their situation from their health professionals (52).

### 4.1 Suicide in RRR communities

Compared to major cities, people living in remote and very remote Australia are 1.7 times more likely to die by suicide (25); with rates for Aboriginals and Torres Strait Islanders nearly twice that of non-Indigenous people (53). Australian men die from intentional self-harm at a rate more than three times that of women (54). Occupation is a risk factor also (55); whereby one farmer dies by suicide every 10 days (56), although a significant risk factor for male farmer suicide appears to be access to firearms (57); a finding supported by a similar study in New Zealand (58). Compounding these elevated risks, is the association between suicide and drought (59). Importantly, reduced access to health services is recognised as a substantial risk factor for increased suicide rates in RRR communities (53). RRR suicide is heterogeneous, suggesting that local, place-based factors may be more important drivers of suicide than mental illness, psychological distress, and poor social and emotional wellbeing (53).

Associations between drought and suicide have also been investigated, with changes in suicide rates being linked with drought condition and specific seasonal timing (59). In addition, disparities exist between suicide rates in RRR and urban Australia; with socioeconomic situation contributing to higher male suicide rates in RRR areas (60). Recently, Hanigan and Chaston (61) estimated the number of suicides attributable to drought to the year 2099 under a range of possible future climate scenarios. They found drought-related suicides increased, across all scenarios, for RRR men aged 10–29 and 30–49 years, highlighting the potential for tragic outcomes for an increased number of families in RRR communities.

### 4.2 Environmental degradation and distress

On an individual level, RRR community members typically have a strong connection to the land and experience a sense of loss when their land becomes degraded (62). A positive correlation between environmental degradation and psychological distress is well established (63), and there is strong evidence that attachment to place in the form of connections with nature are linked to physical and MHWB (64). A recent systematic review, found that environmental degradation and extreme climate events, including drought, were associated with reduced MHWB in RRR communities (65). In particular, people experience distress at the sight of dry paddocks and the loss of ‘green’ amenities such as parks and gardens (66).

### 4.3 Social connection and community led initiatives

Evidence based methods, implemented by RRR community members themselves, have potential to support and maintain MHWB while improving drought resilience. Importantly, Luke et al. (67) found that successful MHWB interventions focused on social connection and self-determination. Better opportunities and encouragement to maintain and develop community connections and social networks can alleviate the MHWB impacts of drought (46). Anecdotally, people from RRR communities talk about scenarios such as losing sporting teams because people cannot afford to play or because so many people have moved out of town. Funding and support are needed to facilitate and maintain activities to preserve RRR residents’ sense of place. Maybery et al. (68) identify schools, sporting and other clubs as important social assets, highlighting the role these organisations play in maintaining connectedness.

## 5 Examples of drought resilience interventions in RRR communities in NSW and QLD, Australia

**Table 1** provides a representative sample of drought resilience interventions targeted at enhancing MHWB. It is by no means exhaustive and, as per feedback obtained in consultations with people from RRR communities, is limited to drought resilience interventions that are easily accessed by RRR community members (i.e. free and available online). Indeed, there is an abundance of such programs, which originate and dissolve rapidly according to funding and political cycles, making it difficult to collate all resources at any given time. Rather, the interventions included highlight commonalities with objectives and lessons learned. Interventions reviewed occur either as targeted programs to deliver services to individuals and communities (targeted services) or larger, all-encompassing networks which in turn support and facilitate the delivery of targeted services (network). The scope of what defines a ‘drought resilience intervention’ is intentionally broad in recognition of the need for interventions to include more than water management, i.e. agriculture, financial situation, MHWB etc. (31, 75). Interventions included in the review range from farm specific initiatives with enterprise focused objectives, through to whole-of-community mental health and wellbeing programs.

**Table 1:** Sample of drought resilience interventions in RRR communities. .

Intervention	Organisation/Funding	Scale	Objective	Activity	Outcomes
Drought Resilience Adoption and Innovation Hubs (69)	Future Drought Fund, Australian Government	Network	Build and support drought-resilience by promoting and maintaining research, development, extension, adoption, commercialisation and knowledge in Australian RRR communities	- Upskilling farmers in entrepreneurship, innovation and commercialisation - On-farm practice and technology trials - training for farmers, e.g. in the use of decision-making tools	- Ongoing - Enhanced collaboration and learning for farmers - Increased connections and drought-resilience for farmers and rural communities
Ifarmwell (70)	NFP entity with external funding from private and industry partners	Service delivery	- Provide evidence-based help to farmers to effectively cope with challenges and stressors	- Free, online intervention based on Acceptance and Commitment Therapy (ACT) informed by research asking farmers what they want - Aimed at both helping those currently experiencing poor MHWB and prevention	- Ongoing - A pre- and post-intervention evaluation found significant decreases in farmers’ distress and increases in MHWB - Users reported high levels of usability and satisfaction, and 94.6% said they would recommend ifarmwell to a friend
NSW DroughtHub (71)	NSW DPI	Network	Provide information and support to farmers to prepare for and manage drought	Three primary programs: Farm Business Resilience Program; Farm Innovation Fund; and Young Farmer Business Program.	- Ongoing - Provides education and support to primary producers
Paddock Between the Ears (72)	Local Land Services (LLS), NSW Government Future Drought Fund, Australian Government	Service delivery	Change mindset around drought preparation and management Invest in social and biophysical capital to build drought-resilience	- Eco-Action sites - Farm Makeover Incentive Program - Hayseeds mentor project - Grazing management field days and workshops	- Ongoing - Development of the Context-Mechanism-Outcome (CMO) framework which is a useful planning tool - Improvements to natural resource assets, drought planning and farm production
Rural Adversity Mental Health Program (73)	NSW Government	Service delivery	Four strategies to link, train, inform and partner with stakeholders	-Information dissemination - Links to resources and support services - Local RAMHP Coordinators	-Ongoing - Production of fact sheets, podcasts, videos and stories
Future Drought Fund’s Networks to Build Drought Resilience (74)	Future Drought Fund, Australian Government	Service delivery	-Provide agriculture dependent communities with resources and opportunities to enhance community and social networks, engagement and wellbeing	-Total of 791 activities completed by 87 community groups	-Completed - 37,841 participants reached - Participants report increased resilience and social capital

The scale column indicated whether the interventions occurred as a targeted program to deliver services to individuals and communities (targeted services) or larger, all-encompassing networks which in turn support and facilitate the delivery of targeted services (network).

Not reported in **Table 1**, because they are intended to encourage face-to-face interactions and therefore are not available online, are less formal interventions such as The Pub Patient nights described in Shenouda (76) and firesheds (13), where the critical yet ‘hard to reach’ populations may be more easily engaged through passive listening. Funding to support these types of social gatherings (i.e. informal drought resilience interventions) are frequently requested during community consultation and can supplement the support offered by online resources and interventions.

### 5.1 What do people want? What interventions work best?

A common theme is the lack of monitoring, evaluation and learning of/from interventions and the need for greater reflection on the specific needs and experiences of residents in RRR communities when developing interventions (77). Results from the few evaluations that have been conducted, demonstrate a lack of validity and reliability (78). The ifarmwell intervention (79) is an example of an evidence-based MHWB resource, fully designed by farmers for farmers, that has undergone robust evaluation (80). Findings from Gunn et al. (80), such as insights into preferred formatting and imagery, level of detail and areas of focus, should be incorporated into tools/resources aimed at improving MHWB in order to ensure the resource is acceptable and feasible to the target audience.

Also reoccurring in the literature and community consultation, is the power of community-led interventions and collective action (30). When the focus is removed from individual wellbeing, a community wellbeing lens can manifest optimism, hope and connection (30). Similarly, an investigation of participatory extension programs (PEPs) in New Zealand, found them effective in providing a dedicated space to talk about MHWB, while linking farmers to support networks (81). PEPs offer an opportunity for balance between the three inextricable factors of farmer MHWB, profitability and sustainability (81). The NFF (47) suggest that farmer MHWB can be supported by encouraging Australian grown produce and products to consumers, advocating for farmer MHWB, increasing funding to interventions and programs, and “checking in” with a mate (67).

## 6 Conclusion

The relationship between drought, MHWB, and drought resilience is complex and interconnected. High drought resilience requires one to be well and have the resources to cope and adapt to stressors and adversity. Policy and community interventions must embrace an understanding of health as determined by place and by an individual’s ability to cope with stressors, to support essential drought resilience, the vehicle that will allow residents from RRR communities to not only survive but thrive. Insights from this paper will inform the development of an online RRR wellbeing toolkit, aimed at improving the MHWB and drought resilience of people in RRR communities.
